# Immunosuppressive Mechanisms of Regulatory B Cells

**DOI:** 10.3389/fimmu.2021.611795

**Published:** 2021-04-29

**Authors:** Diego Catalán, Miguel Andrés Mansilla, Ashley Ferrier, Lilian Soto, Kristine Oleinika, Juan Carlos Aguillón, Octavio Aravena

**Affiliations:** ^1^Programa Disciplinario de Inmunología, Facultad de Medicina, Instituto de Ciencias Biomédicas (ICBM), Universidad de Chile, Santiago, Chile; ^2^Instituto Milenio en Inmunología e Inmunoterapia, Santiago, Chile; ^3^Unidad de Dolor, Hospital Clínico, Universidad de Chile (HCUCH), Santiago, Chile; ^4^Faculty of Medicine, Riga Stradins University, Riga, Latvia

**Keywords:** regulatory B cells, IL-10, TGF-β, IL-35, TIM-1, PD-L1, granzyme B, CD1d

## Abstract

Regulatory B cells (Bregs) is a term that encompasses all B cells that act to suppress immune responses. Bregs contribute to the maintenance of tolerance, limiting ongoing immune responses and reestablishing immune homeostasis. The important role of Bregs in restraining the pathology associated with exacerbated inflammatory responses in autoimmunity and graft rejection has been consistently demonstrated, while more recent studies have suggested a role for this population in other immune-related conditions, such as infections, allergy, cancer, and chronic metabolic diseases. Initial studies identified IL-10 as the hallmark of Breg function; nevertheless, the past decade has seen the discovery of other molecules utilized by human and murine B cells to regulate immune responses. This new arsenal includes other anti-inflammatory cytokines such IL-35 and TGF-β, as well as cell surface proteins like CD1d and PD-L1. In this review, we examine the main suppressive mechanisms employed by these novel Breg populations. We also discuss recent evidence that helps to unravel previously unknown aspects of the phenotype, development, activation, and function of IL-10-producing Bregs, incorporating an overview on those questions that remain obscure.

## Introduction

Over the last two decades, the role of regulatory B cells (Bregs) in immunosuppressive responses has been documented in different contexts and diseases (1). It has been shown, for instance, that Bregs can suppress animal models of autoimmunity, such as experimental autoimmune encephalomyelitis (EAE) (2), collagen-induced arthritis (CIA) (3), and spontaneous colitis (4). Bregs have also been proved to modulate allergy (5), transplantation (6), cancer (7), infections (8), and chronic metabolic diseases (9). Initial studies in the early 2000s attributed this immunomodulation to IL-10, which became the hallmark of Breg suppression (4, 10, 11); however, this notion has lately expanded as new Breg-derived suppressive mediators have been discovered. This review offers to the reader an updated outlook on the immunosuppressive mechanisms employed by Bregs. However, before focusing on the regulatory functions, we will address some concepts with respect to the phenotypic characterization and differentiation of Bregs that still spark some controversy in the scientific community, using IL-10-producing Bregs as a prototypic example.

## The Diversification of Breg Phenotypes

Despite extensive efforts to phenotypically characterize Bregs, including transcriptome analyses and multiparametric flow cytometry, which have been mostly focused on IL-10-secreting B cells, we still lack a definitive set of phenotypic markers or a signature transcriptional regulator (equivalent to FoxP3 in regulatory T cells- Tregs) that enables us to comprehensibly identify Bregs (12–19). This has been further complicated by the large heterogeneity of factors described to induce Bregs *in vivo*. Surface markers such as CD9 (13, 20–22) and TIM-1 (23, 24) have been found to be predominantly but not exclusively expressed across IL-10^+^ B cell populations. Therefore, Bregs remain a functionally defined population based on their capacity to suppress pro-inflammatory responses *in vitro* or *in vivo*, as opposed to effector B cells, which produce pro-inflammatory molecules or induce other cells to do it. According to this consensus, different groups have identified phenotypically distinct B cell populations that, under specific stimulatory conditions, exhibit a superior regulatory proficiency. Many of these populations correspond to discrete developmental stages of the B cell lineage and are enriched in, but not exclusively composed of, cells expressing immunomodulatory factors, such as IL-10.

Mauri and colleagues identified a subset of IL-10-producing B cells with *in vitro* and *in vivo* regulatory capacities among splenic CD21^hi^CD23^hi^CD24^hi^CD1d^hi^ transitional 2-marginal zone precursors (T2-MZP) in mouse (25). Transitional B cells correspond to an intermediate stage between immature cells emerging from the bone marrow and mature cells in the periphery and can be divided into T1, T2, and T3 subpopulations as they progress in their maturation. T2-MZP are T2-stage progenitors committed to differentiate into marginal zone (MZ) B cells in the spleen. Human circulating CD24^hi^CD38^hi^ T2 transitional B cells have also been described to be enriched in IL-10-producing B cells and are able to suppress T cell responses (26). More recently, it has been proposed that other subsets of human transitional B cells, namely, CD24^hi^CD38^hi^CD27^+^ activated memory-like transitional cells, as well as CD24^hi^CD38^hi^IgM^lo^IgD^lo^ anergic-like T3 transitional B cells, also exhibit regulatory properties (27). In addition, murine mature MZ B cells, which lack CD23 expression but maintain high levels of CD1d, have been shown to produce high levels of IL-10 and exert suppressive functions (28).

In parallel, Tedder and colleagues identified a population of murine B cells that express IL-10 after *ex vivo* stimulation with lipopolysaccharide (LPS) plus phorbol 12-myristate 13-acetate (PMA) and ionomycin (P+I); such IL-10^+^ cells are enriched among CD1d^hi^CD5^+^ B cells in the spleen. They termed these cells B10 (29). The human counterpart of B10 was later found to be increased within the CD24^hi^CD27^+^ memory B cell population (30). They proposed that B10 originate from progenitors (B10pro cells) that acquire IL-10-producing competence after stimulation of CD40 or Toll-like receptor (TLR)-4 and have been extensively proven to ameliorate an array of inflammatory conditions upon adoptive transfer (31–37). B10 and B10pro were later found in other B cell compartments, such as murine B-1a cells (31). The B-1 lineage of B cells reside primarily in the peritoneal cavity and spleen and are classified in B1-a and B1-b cells according to the expression of CD5 (38). Peritoneal cavity CD5^+^ B-1a cells are able to secrete high amounts of IL-10 after TLR or CD40 activation and to suppress T cell responses (39–43). Peritoneal cavity and spleen B10 are likely to derive from both the fetal liver and adult bone marrow compartments (42). IL-10^+^ Bregs can already be found among CD1d^lo^CD5^+^ neonatal splenic B cells (31, 44). In addition, a newly identified population of human cord blood CD5^hi^ cells was found to secrete IL-10 upon infection by the respiratory syncytial virus (RSV), leading to inhibition of anti-viral responses and a worse clinical outcome (45). MZ B cells and B1 cells are regarded as innate-like B cells, given their capacity to rapidly respond to innate signals, such as TLR ligands, by producing low-affinity polyreactive natural IgM antibodies and cytokines. Consequently, some authors have denominated those first-line IL-10-producing B cells as innate Bregs; however, the functional implications of this initial regulatory response has not been fully understood (46, 47).

Recently, IL-10^+^ Breg populations have been described among antibody-secreting cells (ASC), such as plasmablasts and terminally differentiated plasma cells, including those populating the bone marrow, in both mice and humans (15, 16, 48–53). Besides, plasma cells expressing other regulatory molecules have also been described, such as IL-35-expressing CD138^+^ plasma cells and PD-L1/PD-L2-expressing IgA^+^ plasma cells (16, 54). These regulatory ASCs are capable of modulating immune responses, as shown in EAE and *Salmonella* infection models (15, 49, 51, 55). It has been observed that murine IL-10^+^ B cells have a greater potential to rapidly differentiate into ASCs than IL-10^−^ B cells, as determined by their upregulation of the ASC fate-determining molecule Blimp-1 and the production of IgM (56). Furthermore, studies in IL-10-reporter mice showed that IL-10^+^ ASCs are already found in naïve mice, while LPS administration or infection with *Salmonella* rapidly expands ASCs that transiently upregulate IL-10 production (15, 43, 49, 56). Subsequent findings in the *Salmonella* infection model showed that in CD138^hi^ plasma cells, the *il10* locus was already primed for transcription (15). Transcriptional analyses of human activated IL-10^+^ B cells have found, as for mouse, the upregulation of genes coding for molecules involved in plasma cell differentiation, such as Blimp-1 and IRF4, thus confirming that human IL-10^+^ B cells are also capable of becoming ASCs (12, 17, 19, 57). Interestingly, it has been suggested that human and mouse IL-10-producing ASCs can be differentiated directly from immature and naïve B cells (15, 55), which is supported by the finding that Blimp-1 is already expressed in recently activated naïve B cells (58, 59).

Altogether, the phenotypical diversity of Bregs suggests that, in addition to classical fate-determining programs, any B cell, regardless of its developmental stage or tissue residence, holds the potential to engage a regulatory program, determined by one or more yet-to-be-identified master transcriptional factors and finely regulated by environmental conditions.

## Breg Differentiation: Nurture or Nature?

B cell regulatory capacity can be triggered by inflammatory cues, such as TLR ligands and pro-inflammatory cytokines, by upregulating a set of inhibitory molecules that restrict the extent of inflammation (20). This idea is supported by findings revealing that T2-MZP IL-10^+^ Breg differentiation can be driven by IL-1β and IL-6, together with CD40 stimulation, in the context of inflammatory arthritis (21). Other pro-inflammatory cytokines involved in Breg induction are B cell-activating factor (BAFF) and A proliferation-inducing ligand (APRIL). BAFF and APRIL are mainly produced by myeloid cells and increased upon inflammation. They participate in B cell homeostasis at different developmental stages, from transitional cell maturation to plasma cell survival. Mice overexpressing BAFF develop spontaneous autoimmunity and increased levels of BAFF have been detected in patients with autoimmune conditions (22). Nevertheless, BAFF and APRIL have been shown to also enhance IL-10^+^ and IL-35^+^ Bregs in mice and humans (23, 24, 51, 60–64).

A regulatory feedback loop has been demonstrated between plasmacytoid dendritic cells (pDCs) and B cells in humans and mice, whereby pDCs respond to inflammation by secreting interferon (IFN)-α, enhancing the production of IL-10 by activated B cells, which in turn suppresses IFN-α production by pDCs (44, 65). Noteworthy, this circuit is impaired in systemic lupus erythematous (SLE), a disease characterized by an IFN-α signature that contributes to pathogenesis by stimulating the differentiation of autoantibody-producing plasma cells. Interestingly, high concentrations of IFN-α in the presence of a TLR9 agonist *in vitro* lead to the differentiation of IL-10^−^ plasma cells instead of IL-10^+^ Bregs (65, 66).

IL-21, together with CD40 ligand (CD40L) and/or TLR9 signals, has been described to boost B10 generation or to drive the emergence of IL-10^+^ plasmablasts during inflammatory processes (53, 67–70). IL-21 and CD40L are expressed by follicular helper T cells (Tfh) in the germinal center (GC), enabling B cells to express affinity mature class-switched antibodies and to become memory B cells or long-lived plasma cells (71). Thus, it is possible that during GC reactions, both effector and suppressive memory B cells and plasma cells are generated. Altogether, these results suggest that during an acute immune response, the generation of effector B cells and Bregs is balanced; however, when inflammation turns chronic, this equilibrium may be lost.

Inflammation-induced IL-10^+^ Bregs employ molecules involved in both mounting and constraining immune responses. CD80 and CD86 are co-stimulatory ligands that enable professional antigen-presenting cells (APCs) to deliver either activating or inhibiting signals to T cells upon binding to CD28 or CTLA-4, respectively (72). Memory and GC B cells exhibit constitutive expression of CD80 and CD86, which can be upregulated following activation. Antigen presentation by B cells has been demonstrated to be required for optimal effector immune responses (73–77). On the other hand, antigen presentation by B cells that lack CD80/CD86 can induce T cell anergy or Tregs (78, 79). Pioneering studies showed that CD86 is involved in B cell suppression of pathogenic T cells and in preventing disease development in a murine model of colitis (80, 81). Similarly, MHC II molecules appear to be necessary for the establishment of cognate interactions between Bregs and activated T cells (57, 70). These results were further confirmed for human IL-10^+^ Bregs, in which CD80 and CD86 work synergistically with IL-10 to suppress Th1 responses (26, 82). Studies in humans and mice have demonstrated that the interaction of CD80/CD86 with CD28 is required for peripheral homeostasis of Tregs, while their engagement of CTLA-4 is important for Treg-mediated suppression (83–85). It was later observed that CD80/CD86-deficient B cells were unable to induce Tregs (81). Although a high expression of CD86 and CD80 have been described in murine and human Breg populations, it appears that their sole upregulation does not enable for a comprehensive identification of Bregs, while a correlation between high expression and functional relevance remains to be fully elucidated (86–88). Therefore, it appears that CD80/CD86 do not constitute a suppressive mechanism by themselves, but instead allow B cells with regulatory capacity to establish cognate interactions with activated T cells to inhibit them or convert them to Tregs.

The fact that, in many cases, similar conditions can induce the differentiation of effector B cells and IL-10^+^ Bregs gives rise to the question of how this fate decision is made. Are some B cells imprinted, at some point during their ontogeny, with the potential to produce IL-10 upon an inflammatory challenge or is the fate of a particular B cell dependent on autonomous perception of spatiotemporal and environmental cues? Is it a stochastic event or is it commanded within narrow ranges of concentration, duration, combination, and concatenation of the stimuli? Is the regulatory phenotype a transient state of B cells, which would die immediately after, or even revert to an effector phenotype if the inflammation persists, or is it a stable trait? Transcriptomic analysis of B cells isolated from arthritic mice showed that the expression of pro-inflammatory cytokines is not substantially different between total IL-10^+^ and IL-10^−^ B cells (14). Lately, a characterization of CpG-stimulated human B cells found that most IL-10^+^ B cells co-express TNF and IL-6 across a broad range of phenotypes. When purified, IL-10^+^ B cells were re-stimulated, their capacity to produce IL-10 was lost, while IL-10^−^ B cells were able to secrete IL-10 after a second challenge, arguing against the existence of a dedicated Breg lineage (18). IL-10^+^ Bregs can also be generated induced by anti-inflammatory factors, such as IL-35 and retinoic acid (89, 90); whether these Bregs are more stable remains unclear.

## Regulation of IL-10 Expression by B Cells

In order to understand better how B cells integrate the different signals that endorse them with the capacity to produce IL-10, we considered relevant to present a general overview of the intracellular pathways involved in this process.

BCR signaling appears to be fundamental for IL-10^+^ Breg development, as mice whose B cells have a transgenic BCR specific for an irrelevant antigen show reduced IL-10 production after *ex vivo* stimulation with LPS and P+I (31). In addition, *in vivo* studies show that suppression of inflammation upon B cell adoptive transfer is significantly more potent when antigen-experienced B cells compared to B cells from naïve mice or B cells specific for unrelated antigens are used, suggesting that BCR engagement is important for optimal IL-10^+^ Breg functions (10, 25, 29, 70, 91). This is further corroborated by the observed expansion or reduction of IL-10^+^ Bregs following CD19 overexpression or deletion, respectively (15, 29, 31). Live-cell imaging of IL-10^+^ Bregs has recently provided evidence that these cells need to capture antigen through their BCR, in order to establish cognate interactions with antigen-specific T cells (52). IL-10-producing murine B cells have shown a diverse, mainly germline-encoded, BCR repertoire, part of which is reactive to self-antigens or antigens from commensal microbes (15, 42, 51, 56). This evidence also supports the notion that Bregs could play a significant role in sensing autoantigens and/or microbiota, limiting a potentially noxious activation of immunity.

Several sources have pointed toward TLR and CD40 activation as an important step in enabling B cells to become competent IL-10 producers. Stimulation of B cells from naïve mice with LPS or CpG (a TLR9 ligand) induces a robust production of IL-10 (31, 92, 93). Moreover, B cell expression of TLR2/4 or their downstream mediator MyD88 is required for an optimal IL-10 production upon LPS stimulation and to achieve *in vivo* suppression by IL-10^+^ Bregs in the EAE and *Salmonella* infection models (31, 49, 92). On the other hand, stimulation of mouse B cells with an agonistic anti-CD40 antibody *in vitro* and *in vivo* leads to an increase in IL-10^+^ Bregs able to suppress arthritis and lupus in murine models (3, 94), while B cells overexpressing CD40L exhibit higher frequencies of B10 (31). The activation of CD40 is believed to be important for IL-10^+^ Breg generation as part of cognate interactions with activated T cells, as B cells lacking CD40 are unable to inhibit T cell activation and to protect mice from EAE or colitis (10, 70, 80). Human B cells were also shown to produce IL-10 in response to TLR ligands and CD40L (30, 55, 95). Innate immune cells, such as pDCs, mast cells, and type 3 innate lymphoid cells, can be additional sources of CD40L-derived signals (65, 96–98).

B cells express a large set of inhibitory receptors that deliver negative signals to counterbalance their activation. Some of these receptors have also been found to restrain IL-10 production by Bregs. CD22 is an inhibitory receptor responsible for activating phosphatases upon binding to mammalian-restricted sialylated proteins, in order to dampen BCR activation by self-antigens (99). In agreement with CD22 inhibitory function, murine B10 lacking CD22 present an increased production of IL-10 after short-term LPS stimulation (31). Likewise, a dramatic expansion of IL-10^+^ Bregs is observed when CD22 deletion is incorporated in CD40L-overexpressing B10 after TLR or CD40 activation, and they exhibited enhanced EAE suppressive capacity (31, 100). However, lack of CD22 impairs the survival of regulatory peritoneal B-1a cells and their recruitment to lymphoid organs (101). Much less studied is the role on B cells of CD72, a receptor for the inhibitory ligand semaphorin-4D, but that also recognizes RNA-containing self-antigens, downregulating BCR signaling (102). *Cd72*^−/−^ mice display an increased number of LAG-3^+^CD138^hi^ plasma cells, which produced augmented IL-10 levels following *Salmonella* infection, leading to impaired control of the bacteria (15).

Although antibody-mediated crosslinking of the BCR precludes IL-10 production upon simultaneous stimulation with LPS or CD40 ligation in B cells from naïve mice (29, 31, 42, 94) or human blood (30, 103), Bregs from mice with induced autoimmunity are able to secrete IL-10 when challenged with cognate antigens, or BCR crosslinking antibodies, plus CD40 ligation (3, 10, 25). It is possible to infer that a sequential integration of signals (inflammatory signals first, followed by T cell help in the form of CD40L and IL-21, plus repeated antigenic stimulation) can determine the acquisition of IL-10^+^ Breg proficiency *in vivo* (32).

The PI3K-Akt pathway, downstream of BCR engagement, is critical for B10 development in mice, as well as for IL-10 production following TLR4 or CD40 stimulation, suggesting that a tonic signaling through the BCR is required for further induction of IL-10 expression by other routes (88). In accordance with this idea, perturbations in the BCR signalosome adaptor BLNK and downstream kinase Btk have been found to curb LPS-mediated activation of the transcription factor STAT3 and ensuing IL-10 production by murine B10 (104). TLR stimulation followed by BCR crosslinking has been shown to induce IL-10 expression by B10 from naïve mice, by the calcineurin-dependent nuclear translocation of the transcription factor NFAT, triggered by store operated Ca^2+^ influx (105). This emphasizes the idea that pre-sensitization of B cells with innate signals is a pathway to acquire a full Breg competence. An NFAT-independent calcineurin-mediated induction of IL-10 production in TLR-activated B cells has also been described (106). Of note, it has been shown that NFAT-dependent IL-10 production in B cells involves IRF4 binding to the *il10* locus, suggesting a link between the induction of IL-10^+^ Bregs and later development of plasma cells (55, 107). Recently, Blimp-1 has been described to direct the differentiation of IL-10^+^ plasma cells (108, 109); however, further research is needed to dissect the pathways leading to this particular phenotype.

As for TLR-induced signals, CD40-mediated STAT3 activation appears to be central for IL-10 expression on B cells. STAT3 phosphorylation is increased in CD40-activated transitional B cells of healthy subjects but not in those from SLE patients (26). In addition, CREB, p38 MAPK, ERK1/2, and PI3Kδ have been described to be required for IL-10 upregulation following TLR and/or CD40L stimulation (58, 67, 110). c-Maf, a well-known transactivator of IL-10 in various cell types, has also been shown to mediate IL-10 transcription by LPS-stimulated murine B cells (111). Interestingly, c-Maf interacts with aryl-hydrocarbon receptor (AhR) in promoting IL-10 expression on Tr1 cells (112). Recent studies have confirmed AhR as a critical factor governing TLR and BCR-mediated IL-10 production in CD19^+^CD21^hi^CD24^hi^ B cells, while inhibiting the expression of pro-inflammatory molecules and the differentiation of GC B cells and plasma cells (14, 113).

AhR activation in Bregs requires increased levels of a serotonin metabolite, 5-HIAA, generated by gut commensal bacteria that are enriched after treatment with the short-chain fatty acid (SCFA) butyrate (113). Gut microbiota promotes the differentiation of IL-10^+^ T2-MZP Bregs in arthritic mice by inducing IL-6 and IL-1β production by gut-associated lymphoid tissue (GALT) DCs and macrophages, implying that during inflammation, T2-MZP migrate to the GALT to acquire the necessary signals to become Bregs, a process that appears to be dependent on AhR expression (14, 21). These results are in line with evidence showing that skewing the gut microbiota composition with oral antibiotics or probiotics can modify IL-10^+^ Breg frequencies (114–116). Moreover, gut commensal flora-specific IgA^+^IL-10^+^ ASC can be recruited to the brain of EAE mice and reduce disease severity (51). Although gut-associated bacteria have been reported to be dispensable for IL-10^+^ Breg development in naïve mice (31, 42), current evidence suggest that their presence might be critical for the development of both inflammation and inflammation-induced IL-10^+^ Bregs (21).

A direct effect of SCFA pentanoate in potentiating CpG-induced IL-10 production by B cells was reported to be dependent on the activation of p38 MAPK and the glycolytic pathway, enhancing the phosphorylation of mTOR and its downstream targets S6K and ribosomal S6 (117). An induction of IL-10 by an alternative pathway involving ERK1/2 and RSK, upstream mediators of S6 phosphorylation, has been described in LPS-stimulated peritoneal B cells (118). mTOR inhibition precludes IFN-λ and IFN-α-induced IL-10 production in BCR-activated human B cells (119). In agreement with these results, the levels of IL-10-producing B cells are reduced in kidney transplant patients receiving inhibitors of mTOR (120). Another key regulator of the glycolytic pathway, the enzyme GSK3β, has been described as a repressor of IL-10 synthesis in B cells by enhancing the expression of NFATc1 (121). mTOR also enhances the activation of PPAR-γ, a key transcription factor for adipogenesis and metabolic reprogramming in activated T cells (122). Remarkably, PPAR-γ-deficient B cells present a reduced IL-10 production and impaired suppressive capacities, while treatment with a PPAR-γ agonist significantly expanded IL-10^+^ B cells in high fat diet-fed mice (123, 124). Furthermore, free fatty acid palmitate can increase survival and IL-10 synthesis by adipose tissue-resident B cells, suggesting a potential participation of lipid metabolism in Breg functions (125). Accordingly, a publication has recently described that atorvastatin, an inhibitor of cholesterol metabolism, prevents IL-10 production by CpG-activated B cells through reducing geranylgeranyl pyrophosphate-mediated activation of Akt and inhibition of GSK3β, leading to a decreased transcription of Blimp-1 and IL-10 (58). The metabolic regulation of B cell responses is an emerging field that would offer new insights into the factors that govern the fate of activated B cells, including Bregs.

As mentioned above, inflammatory cytokines potentiate IL-10 expression in B cells. For instance, IL-6 and IL-1β are able to prolong the phosphorylation of NF-κB and STAT3 achieved by CD40 ligation in mouse B cells (21). Likewise, IL-21 and APRIL increase phosphorylation of STAT3 and subsequent IL-10 production by B cells (53, 60, 67, 68). It has been observed that IFN-α boosts TLR7/8-induced IL-10 production by human B cells by enhancing the phosphorylation of ERK1/2 and STAT3 (66). IFN-α-induced STAT3 phosphorylation is decreased in B cells from SLE patients and is restored upon successful B cell depletion therapy (65). Contrastingly, the role of IFN-γ on IL-10^+^ Bregs is still controversial (70, 126, 127). IFN-γ upregulates IL-10 production induced by TLR7/8 and TLR9 agonists in mouse and human B cells, and this involves protein kinases p38 and JNK (118). On murine MZ B cells, IFN-γ, BCR, and TLR9 signals converge for the prolonged activation of the transcription factor CREB to induce IL-10 expression (128, 129). A recent publication reported that human IL-10^+^ B cells distinctively express TNF receptor 2 (TNFR2) following TLR9 stimulation and that they respond to TNFR2 agonists by increasing their IL-10 production. This gives rise to the possibility that interaction of Bregs with membrane-bound TNF on activated T cells or monocytes may influence their suppressive functions (130).

Although autocrine IL-10 appears not to be necessary for the development of B10 or IL-10^+^ ASCs (56), an autocrine effect on IL-10^+^ Breg expansion following stimulation has been suggested (131). Newly discovered IL-35 also increases IL-10 production in TLR-stimulated human and mouse B cells by triggering STAT3 activation (132). Moreover, it has been recently described that tonsil-derived mesenchymal stem cells (MSCs) are an important source of IL-35, which upon co-culture with murine B cells, promote the expansion of B10, providing further evidence for the therapeutical use of MSCs in autoimmune diseases or organ transplantation (133). Indeed, inoculation with MSCs has been described to expand IL-10^+^ Bregs in EAE (134), murine colitis (135, 136), graft vs. host disease (GVHD) (137, 138), and allograft transplantation (139–141).

Other cytokines and factors described as IL-10^+^ Breg inducers include IL-33 (142, 143), granulocyte macrophage colony-stimulating factor (GM-CSF) (144), a GM-CSF/IL-15 fusokine (57), thymosin-α1 (145), human chorionic gonadotrophin (146–148), retinoic acid (149), hypoxia-inducible factor-1α (150), vitamin D3 (90), insulin-like growth factor 2 (151), and indoleamine 2,3 deoxygenase (IDO) (134). Conversely, IL-10 production by B cells has been determined to be repressed by several molecules such as soluble TNF (152, 153), transforming growth factor (TGF)-β (70, 126, 127), IL-4 (154, 155), IL-13 (156–158), IL-17 (159), lipoxin A4 (160), prostaglandin E2 (161), and progesterone and estradiol (162), although some results are divergent, likely due to different stimulation conditions or subpopulations assessed (163, 164). In addition, IL-10 expression by B cells can be upregulated by neurotransmitters such as dopamine (165) and norepinephrine (166), as well as NMDA-receptor antagonists (167). Despite lack of information about transcriptional repressors of IL-10, it has been reported that the transcription factor Foxd3 negatively regulates the expression of IL-10 in LPS-stimulated mouse B cells (168).

Despite extensive research into the epigenetic regulation of IL-10 expression in immune cells, there is still a paucity of studies on B cells (169, 170). Recent work explored the DNA methylation signature of the *il10 locus* in B cells and defined “early” and “delayed *il10* regulatory regions,” which are demethylated, or accessible, in B cells that produce IL-10 after short- or long-term stimulation with LPS, respectively (171). These results are even more meaningful considering that the “delayed *il10* regulatory region” is a binding site for IRF4 in IL-10^+^ plasmablasts, where it might interact with NFATc1 to promote IL-10 expression (55).

Chromatin accessibility analysis of murine IL-10^+^CD21^hi^CD24^hi^ B cells has shown open chromatin regions in the *il10 locus*, as expected, but also in the *Ahr locus*, indicating an active transcription of this key factor (14). Of note, butyrate supplementation, which activates AhR in such CD21^hi^CD24^hi^ B cells, increases accessibility at binding motifs for partner transcription factors of AhR, which can be attributed to butyrate activity as histone deacetylase inhibitor, as has been shown for Tregs and total B cells (113, 172). A short isoform of NFATc1, NFATc1/αA, has been shown to repress IL-10 transcription in B cells, in part by binding to the histone deacetylase HDAC1 upon BCR or TLR activation (173, 174). TNF and IL-13 repression of IL-10 production by human B cells can be partially mediated by enhancing HDAC11 expression (153, 156). Histone acetylation, a critical mechanism in increasing chromatin accessibility, is read by BET proteins, which facilitate chromatin remodeling (175). Although this territory remains largely unexplored in Bregs, it was recently reported that BRD4 BET enhances IL-10 expression in LPS-stimulated murine B cells by associating with NF-κB on the *il10* promoter (176).

Finally, the regulation of IL-10 expression by small, conserved, non-coding RNA—microRNA (miRNA)—has been evaluated in recent years. Initial transcriptomic studies found 77 differentially expressed miRNAs in mouse B10 compared to non-B10, the impact of which has not yet been explored (13). However, the function of a handful of miRNAs in modulating IL-10 transcription in mouse and human B cells has been described, many of which are responsive to pro- and anti-inflammatory cytokines and have been found to be dysregulated in a variety of immune-related disorders (154, 155, 157–159, 177–189). Many other signaling molecules, as well as epigenetic and post-transcriptional regulators of IL-10 expression in B cells, have been proposed; however, a definite transcriptional program remains obscure (170).

## Suppressive Mechanisms of Bregs by Soluble Molecules

**Figure 1:**
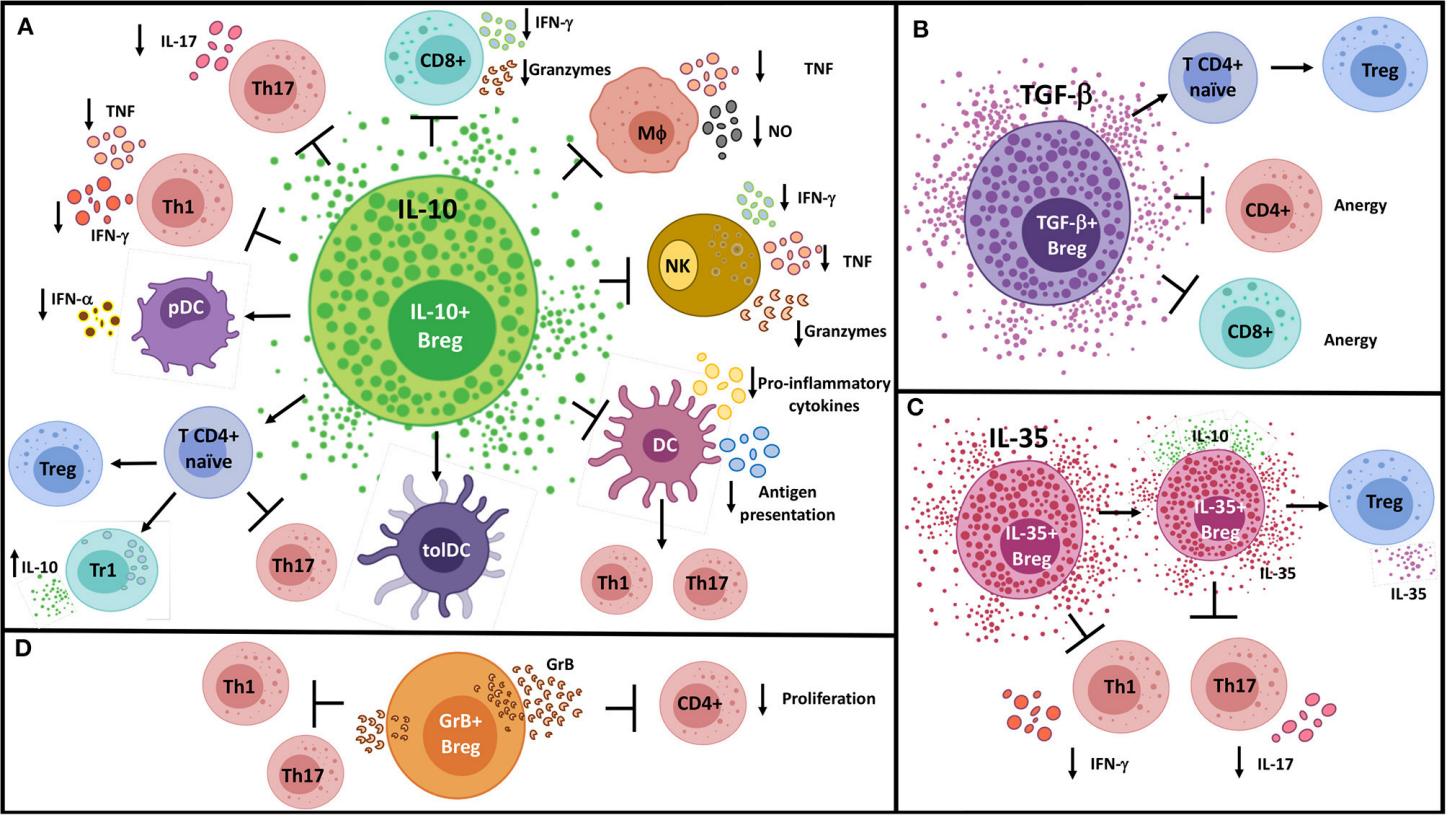
Suppressive mechanisms of Bregs by soluble molecules. **(A)** IL-10^+^ Bregs inhibit Th1, Th17, and CD8^+^ T cell responses; convert naïve CD4^+^ T cells into regulatory T cell populations; and modulate pro-inflammatory innate cells through the production of IL-10. **(B)** Likewise, TGF-β^+^ Bregs operate on naïve CD4^+^ T cells to generate FoxP3^+^ Tregs, in addition to induce anergy in CD4^+^ and CD8^+^ T cells. **(C)** IL-35^+^ Bregs can promote “infectious tolerance” by inducing IL-35-producing Tregs and expanding the generation of IL-35^+^ Bregs. **(D)** GrB^+^ B cells have been shown to inhibit Th1 and Th17 cell responses and to reduce CD4^+^ T cell proliferation by degrading the TCR ζ-chain. DC, Conventional dendritic cell; GrB, Granzyme B; MΦ, Macrophage; NK, Natural killer cell; NO, Nitric oxide; pDC, Plasmacytoid dendritic cell; TolDC, Tolerogenic dendritic cell; Tr1, Type-1 regulatory T cell.

The ability of B cells to secrete immunomodulatory molecules has gained increasing attention over the last years. Although many Breg functions can be attributed to the release of anti-inflammatory cytokines, other soluble molecules have been recently described to mediate B cell suppression, such as granzyme B (GrB) (190), adenosine (191–193), IDO (194), progesterone-induced blocking factor 1 (195), and heat shock protein-70 (196). The main suppressive mechanisms employed by Bregs through the secretion of soluble molecules are summarized in Figure 1.

### IL-10

*In vitro* and *in vivo* adoptive transfer experiments initially showed that different populations of murine-activated B cells are able to suppress antigen-specific CD4^+^ T cell proliferation and pro-inflammatory cytokine production in an IL-10-dependent manner and reduce inflammation in autoimmunity models, such as CIA (3, 23, 25, 36, 197), antigen-induced arthritis (11, 14, 21, 113), spontaneous lupus (94), type 1 diabetes (198), colitis (42), and EAE (10, 57, 70, 105, 199). IL-10^+^ B cells and plasma cells have been found to gain access to the central nervous system and suppress pathogenic CD4^+^ T cells, demonstrating their ability to act *in situ* (51, 200, 201). The inhibition of T cell proliferation and production of Th1 cytokines (IFN-γ and TNF) was also demonstrated in *in vitro* studies involving human B10 and IL-10^+^ transitional B cells; however, B cells from patients with autoimmune diseases such as SLE, RA, and type 1 diabetes are unable to achieve this suppression (26, 30, 67, 95, 202, 203). Moreover, immunocompromised humans or mice with Wiskott–Aldrich syndrome protein deficiency (204–206), as well as patients with common variable immunodeficiency (207, 208) exhibit low numbers of IL-10^+^ B cells that correlate with increased pro-inflammatory CD4^+^ T cell responses. As T cells express low levels of IL-10R, it has been suggested that the CD4^+^ T cell suppression relies on an autocrine effect of IL-10, decreasing CD86 expression and co-stimulation potential of B cells (209, 210). Murine and human Bregs can also act on naïve CD4^+^ T cells, blocking their differentiation into Th17 cells, a feature important for recovery from CIA. This pathway is also defective in RA patients (36, 211).

Pro-inflammatory CD4^+^ T cell responses are a critical driving force of chronic cardiovascular diseases (212). In the spleen of atherosclerosis-prone mice, increased IL-10^+^ B cells have been detected. These B cells were able to suppress Th1 responses *in vitro* in an IL-10-mediated fashion (213). In a model of arterial injury, a high number of IL-10^+^ T2-MZP were found in lymph nodes and, upon adoptive transfer, were able to reduce atherosclerotic lesions (9). IL-10^+^ Breg-mediated atheroprotection has been suggested to be dependent on angiotensin II signaling in B cells (214), L-selectin expression (215), and IDO activation (216). IL-10^+^ B cells have also been recovered from tertiary lymphoid organs in arteries of atherosclerotic mice, suggesting that in atherosclerosis (like in neuroinflammation), Bregs may also suppress inflammation *in situ* (217). In addition, scattered publications have reported reduced levels of transitional or IL-10^+^ B cells in peripheral blood of atherosclerotic patients compared to healthy controls (154, 178, 218, 219). Furthermore, there is accumulating evidence pointing toward a protective role of IL-10-producing B cells in controlling inflammation and tissue damage following experimental ischemic stroke (220–225). A recent study exploring the involvement of IL-10-producing B cells in myocardial infarction in rodents observed that these cells are recruited to the infarcted heart from pericardial adipose tissue and contribute to limit the extent of inflammation-related injury (226). In this regard, IL-10-producing B cells have been found to reside in subcutaneous and visceral adipose tissues from mice and humans; in diet-induced obesity models, they contribute to the restriction of local inflammation and thereby control insulin resistance (125, 227, 228). Furthermore, adipose tissue or peripheral blood IL-10^+^ B cells are numerically and functionally altered in both obese patients and individuals with type 2 diabetes (229–234).

In addition to inhibiting immunogenic CD4^+^ T cell populations, IL-10^+^ Bregs are also able to convert naïve CD4^+^ T cells into Tregs and IL-10-secreting type-1 regulatory CD4^+^ T cells (Tr1), as shown in experimental arthritis (11, 14, 197), lupus (34, 94), and EAE (104, 235). In agreement with this evidence, human IL-10^+^ Bregs from healthy individuals have been shown to induce Tregs and Tr1 cells; these mechanisms are impaired in autoimmune patients (211, 236, 237), but enhanced in chronically virus-infected patients (238).

IL-10^+^ Bregs promoting a shift toward a Th2 response has been demonstrated to be beneficial in EAE (115, 239–241), CIA (25), and lupus (94). IL-10^+^ Bregs have also been shown to confer susceptibility or protection to parasite-derived immunopathology in mice, depending on the pathogen, partially due to their ability to skew the immune response in favor of Th2 immunity (242–246). An expansion of IL-10^+^ Bregs has been detected in patients with parasite infections (247, 248). Moreover, parasite-induced IL-10^+^ Bregs have been suggested to alter the course of multiple sclerosis (MS) in humans (249) and to alleviate disease in allergy models (250–254). Remarkably, allergen-specific oral tolerance induction in mice was associated with increased frequency of IL-10^+^ Bregs in mesenteric lymph nodes (255), and allergen specific immunotherapy drove an increase in IL-10-producing B cells in allergic patients (17, 256, 257). These findings are validated by studies showing that adoptive transfer of IL-10^+^ B cells or plasma cells not only suppresses Th2 and/or Th17 responses but also induces Tregs, in allergic airway inflammation (247, 251, 254, 258–261) and food allergy (255).

The induction of tolerance to exogenous antigens by IL-10^+^ Bregs has been further explored in the field of allograft transplantation. Studies in a model of hematopoietic stem cell transplantation (HSCT) demonstrated that both donor and host IL-10-producing B cells are required to prevent acute and chronic GVHD, in part due to the suppression of T cell cytokine production and induction of Tregs (37, 116, 262–264). Similarly, a protective role of IL-10^+^ Bregs in human GVHD has been suggested after higher frequencies of these cells were found reconstituting recipients of HSCT who developed tolerance, compared to those who developed GVHD (53, 138, 265, 266). Analogous evidence has been obtained from solid organ transplantation models. Transitional splenic B cells prolonged skin allografts upon adoptive transfer and suppressed allo-specific CD4^+^ T cells *in vitro*; this effect was dependent on IL-10 and galectin-1 expression in donor B cells (114, 267). IL-10 production is critical for T2-MZP-mediated cardiac allograft tolerance by downmodulating Th17 responses (268, 269). In the clinical field, several studies have pointed out that low numbers or impaired suppressive functions of transitional B cells are associated with a higher risk of kidney graft rejection (270–272). In addition, IL-10^+^ B cells have been shown to be increased in patients tolerating lung transplants compared to patients developing chronic graft dysfunction (273). Another study observed that the IL-10^+^/TNF^+^ ratio in T1 transitional B cells was lower in patients rejecting kidney grafts compared to patients with stable graft function, and predicted a worse long-term outcome (274, 275). A number of studies have also shown increased circulating transitional B cells in tolerant kidney-transplanted patients despite withdrawing immunosuppression, compared to patients with stable grafts on immunosuppression (276–279). This high B cell-derived IL-10 expression was later associated with a decreased expression of CD86 and an increased CD40 activation by recipient T cells (209, 280). Besides allograft tolerance, mounting evidence implicates IL-10^+^ B cells in mechanisms governing tolerance to semi-allogeneic fetus during pregnancy in both mouse (147, 281–285) and human (146, 147, 286–289), which include the generation of tolerogenic DCs and Tregs and a reduction in Th17 cells (281, 282, 290).

In addition to their effects on CD4^+^ T cells, it has been demonstrated that IL-10^+^ Bregs inhibit antigen-specific CD8^+^ T cell activation. For instance, during murine cytomegalovirus and influenza virus infections, B cell-derived IL-10 restrains virus-specific CD8^+^ T cell responses (43, 291). Breg-mediated inhibition of CD8^+^ T cell proliferation and IFN-γ production has also been demonstrated in patients infected with human immunodeficiency virus (HIV) (292, 293) and hepatitis B virus (294) and in a humanized murine model of hepatitis C virus infection (295). There is evidence that Bregs can reduce CD8^+^ T cell responses indirectly through inhibition of CD4^+^ T cell help (296). Thus, the activity of IL-10^+^ Bregs generated during chronic viral infections can become dysregulated, impairing virus clearance. However, in other settings, such as ankylosing spondylitis and obesity, defective IL-10-mediated downregulation of CD8^+^ T cell responses by B cells may promote unwanted inflammation and tissue damage (125, 297).

Given the pleiotropic effects of IL-10 on B cells, it has been difficult to ascertain how IL-10^+^ Bregs modulate humoral immune responses. IL-10 is a potent survival factor for B cells, preventing GC B cell apoptosis and favoring their development into plasma cells (298–302). *In vitro* and *in vivo* studies have proposed that these actions can be provided through autocrine and paracrine IL-10 secretion (19). In addition, IL-10^+^ Bregs can suppress IL-10^−^ B cell proliferation (142). Therefore, while suppressing T cell activation, B cell-derived IL-10 may support a sustained humoral response, involving their own progression toward IL-10^+^ ASC.

The regulation of innate elements of the immune response appears to be a fundamental feature of IL-10^+^ Bregs. B10 were shown to inhibit TNF or nitric oxide (NO) production by monocytes, macrophages, and microglia (30, 200, 303). Besides, murine IL-10^+^ Bregs can prevent the recruitment of neutrophils to the sites of inflammation in a model of colitis (42). Likewise, in a model of *Salmonella* infection, IL-10 production by B cells was essential for reducing neutrophil mobilization and TNF production, as well as downmodulating NK responses (49); this ultimately contributes to an impaired pathogen clearance, as has been shown for other bacterial or fungal infections (109, 304). B cell-derived IL-10 has also been suggested to be important in increasing the turnover of maturing neutrophils in the bone marrow following *Pneumocystis* infection in mice (305). Additionally, murine Breg-derived IL-10 can suppress IgE-mediated degranulation of mast cells (97). As mentioned above, human and mouse IL-10^+^ Bregs are potent inhibitors of cytokine production by pDCs, but also by conventional DCs (44, 65).

Since DCs are conspicuous for their capacity to bridge innate and adaptive immune responses by priming antigen-specific naïve T cells, it has been evaluated whether IL-10^+^ Breg suppression of T cell responses can be indirectly mediated through DCs. Matsushita et al. found that co-incubation of murine DCs with activated B10 reduce DCs ability to activate encephalitogenic CD4^+^ T cells (199). This evidence, together with data showing that IL-10^+^ B cells or plasmablasts inhibit Th1 and Th17-inducing DCs or induce tolerogenic DCs, in addition to direct inhibition of Th1 and Th17 cells, contribute to clarify the suppressive role of B10 in EAE and other inflammatory conditions (55, 92, 306, 307). Similarly, B cell-mediated induction of Th2 responses in *Leishmania* infection is associated with IL-10-dependent decrease in IL-12 production by DCs (244). Modulation of antigen presentation by IL-10^+^ Bregs is also frequent during bacterial infections. For instance, *Escherichia coli* induces a population of IL-10^+^ Bregs capable of inhibiting DCs maturation (308). In experimental infection with *Listeria*, B10 decrease phagocytosis of bacteria and subsequent IFN-γ and TNF secretion by macrophages, reducing antigen-specific CD4^+^ T cell proliferation and cytokine production (309). Another study showed that MZ B-derived IL-10 inhibits NO synthesis on neighboring metallophilic macrophages, increasing the intracellular survival of *Listeria*, and facilitating the trans-infection of DCs, which leads to increased bacterial burden, but also to efficient CD8^+^ T cell priming (310). Interestingly, it has been shown that IL-10^+^ Bregs can establish longer contact times with CD4^+^ T cells than their IL-10^−^ counterparts (11). Based on this, a supplementary mechanism for interrupting antigen presentation has recently been suggested, where longer cognate interactions between IL-10^+^ Bregs and T cells reduces the chances of effector T cells to encounter antigen-loaded DCs and become activated (52). The effects of murine IL-10^+^ Bregs on DCs can be extrapolated to humans, as human B cells overexpressing IL-10 have been described to suppress differentiation of monocytes to DCs and to promote the generation of tolerogenic DCs (311).

Immune evasion processes involving IL-10^+^ Bregs can also be induced by neoplastic cells. The role of Bregs in cancer had already been suggested in experimental models, since mice lacking or depleted of B cells exhibit enhanced anti-tumor CD8^+^ T cell responses and are resistant to tumor progression (312–316). More recently, IL-10^+^ B cells and IL-10^+^ plasmablasts have been recovered from tumor or tumor draining lymph nodes, while cancer patients show increased circulating IL-10^+^ Bregs (82, 317–323). Tumor cells can induce B cells to secrete to IL-10 through multiple mechanisms including CD40L signals (315, 322) and tumor-derived exosomes (324–327). *In vitro* and *in vivo* studies have demonstrated that IL-10^+^ Bregs induced by mouse tumor cells are able to reduce IFN-γ production by antigen-specific CD8^+^ and CD4^+^ T cells as well as NK activation (315, 324). Adoptive transfer of B10 can also prevent anti-CD20-mediated lymphoma depletion, likely through inhibiting macrophage activation (303). In a similar manner, tumor-induced human IL-10-producing B cells or plasmablasts have been described to suppress IFN-γ and GrB expression by CD8^+^ T cells (317, 318, 321), as well as pro-inflammatory cytokines secreted by CD4^+^ T cells (82, 324, 328). These effects can have an impact on tumoral cell survival, as demonstrated by a study where IL-10^+^ Bregs showed the ability to block antibody-dependent cytotoxicity of NK on myeloma cells (329). These results have prompted the design of new therapeutic tools for cancer based on specific IL-10^+^ Breg-depletion, as recently published (330, 331).

In many cases, activated B cells can simultaneously produce other anti-inflammatory cytokines along with IL-10, such as TGF-β and IL-35, which can be uniquely responsible for some of the immune regulatory properties assigned to Bregs, as described below.

### TGF-β

The members of TGF-β superfamily exhibit pleiotropic activities, the effects of which are both cell type- and context-dependent. TGF-β participates in the regulation of B cells at various stages of their development, with an important involvement in the control of self-tolerance and autoimmunity (332). On the other hand, evidence confirms that resting human B cells express TGF-β and TGF-β receptors, the expression of which is increased upon activation (333). It has been extensively described that several murine and human IL-10^+^ Breg populations can also secrete TGF-β; however, in many cases, the role of TGF-β suppression mediated by these cells has been excluded (26, 94, 334, 335). Nevertheless, in other cases, TGF-β has been described to exert a dominant part in Breg functions. In particular, TGF-β has the capacity to convert naïve CD4^+^ T cells into Tregs (336). Consequently, it was not surprising to find out that TGF-β^+^ Bregs are able to induce Tregs in healthy mice and humans (194, 337), as well as in inflammatory conditions such as transplantation (338), allergy (339, 340), and cancer (86, 341–343). In this regard, studies in a breast cancer model have described a population of CD25^hi^CD69^hi^ Bregs, induced by tumor-secreted factors such as leukotriene B_4_. These Bregs promote lung metastasis by inducing Tregs *via* STAT-3-dependent TGF-β production (86, 342, 344, 345). In addition, TGF-β secreted by tumor-evoked Bregs increased reactive-oxygen species and NO production by myeloid-derived suppressive cells, which in turn inhibited proliferation of CD4^+^ and CD8^+^ T cells, favoring metastasis (346).

Moreover, in accordance with the inhibitory functions of TGF-β on effector T cell proliferation and differentiation, TGF-β^+^ Bregs were reported to trigger anergy in CD4^+^ and CD8^+^ T cells (347, 348). In a more recent study, it was detailed that transgenic mice deficient for TGF-β1 specifically in B cells developed EAE at an accelerated rate compared to wild-type controls. This was associated with an increased frequency of activated DCs and an expansion of pathogenic IFN-γ^+^ IL-17^+^ CD4^+^ T cells in the central nervous system, suggesting indirect control of inflammatory T cells by TGF-β^+^ Bregs (349). TGF-β-producing Bregs can also be induced in mice infected with helminths. Such Bregs are able to suppress Th1- and/or Th2-mediated colitis through a mechanism involving cooperation with anti-inflammatory macrophages (350). Noteworthy, TGF-β^+^ Bregs have been described to be decreased in the alveoli of RA patients with interstitial lung disease (351), and in the blood of patients with myasthenia gravis (352), and increased in the blood of gastric cancer patients (341).

The phenotype of TGF-β^+^ Bregs has been poorly characterized. Some reports have identified them within the CD5^+^ or CD25^hi^CD69^hi^ B cell populations in mouse (86, 353) and within the T2 transitional B cell subset in humans (341).

### IL-35

IL-35, a potent anti-inflammatory cytokine, is the newest member of the IL-12 family of heterodimeric cytokines and is composed of the Ebi3 and the IL-12p35 chains. IL-35 has been described to be produced in large quantities by mouse and human Tregs and to be an important factor for their suppressive activities (354). Similarly, a role for IL-35 in Treg induction has been proposed; these Tregs then further mediate suppression *via* IL-35 (355). However, whether other immune cell types can produce IL-35, and what functions this cytokine exerts, is a matter that had not been fully assessed until recently.

In this regard, Egwuagu and colleagues have shown that addition of IL-35 to LPS-stimulated human or murine B cells induces not only an expansion of IL-10^+^ Bregs but also the generation of IL-35-producing B cells (89, 356). These cells, named IL-35^+^ Bregs, develop spontaneously in mice with experimental autoimmune uveitis (EAU), exhibit a CD1d^hi^CD21^hi^ phenotype, and are a major source of IL-35. Furthermore, IL-35^+^ Bregs are expanded *in vivo* upon injection of IL-35, which is associated with an increase in Tregs, and a decrease in Th1 and Th17 cells *via* IL-10 and IL-35 production, reducing the severity of EAU (89, 356), but impairing protective immunity in a mycobacterial infection model (357). In parallel, Shen et al. described the ability of CD40 and TLR4 stimulation to induce IL-35 production by murine B cells. They also reported that a population of CD138^+^ plasma cells were the main producers of IL-35 in EAE mice and mice infected with *Salmonella*. B cell-restricted deletion of either IL-35 chain exacerbated EAE, but reduced *Salmonella* burden. These phenomena were correlated with exacerbated Th1 and Th17 responses and higher antigen-presenting capacity of B cells (16). The generation of IL-35^+^ Bregs appears to be mediated by the IL-12p35 subunit and IL-12Rβ2, leading to the activation of STAT1, STAT3, IRF-4, IRF-8, and BATF (89, 147, 356, 358). Whether IL-35^+^ and IL-10^+^ Bregs correspond to separate populations or display some degree of overlap is an issue that requires further clarification.

In a model of pancreatic ductal adenocarcinoma, IL-35^+^ Bregs were found to be induced by IL-1β plus IL-6 and CD40 stimulation and to participate in tumorigenesis (359). These results are in line with a previous study showing that IL-35^+^CD1d^high^ B cells are expanded in mouse and human pancreatic tumors and that these cells are able to promote tumor growth *via* IL-35 secretion (360). IL-35^+^ Bregs can also be generated from MZ B cells upon exposure to BAFF through the classical NF-κB pathway. These cells are increased in the spleen of mice with lupus and are able to suppress Th1 responses and expand Tregs in an IL-35-dependent manner (62). IL-35^+^ Bregs have been detected in human decidua (361), while low frequencies have been found in decidua of abortion-prone mice (362). IL-35^+^ B cells have also been detected in intestinal mucosa from patients with Crohn's Disease (CD), but not from ulcerative colitis (UC) (363). Although IL-35 expression by *in vitro*-activated peripheral blood B cells is defective in CD patients, it can be rescued after incubation with exogenous IL-35, endowing them with enhanced suppressive capacities on pathogenic Th1 and Th17 cells (364). Similarly, Breg-mediated suppression of Th1 and Th17 responses in UC patients was restored upon addition of IL-35 (365), supporting the therapeutic use of IL-35 for inflammatory bowel diseases. Reduced frequency of IL-35^+^ Bregs in the peripheral blood has been observed in SLE patients (366), while increased frequencies of these cells have been reported in patients with active tuberculosis (367), leprosy (368), and gastric cancer (369). This further broadens the spectrum of conditions that could benefit from IL-35^+^ Breg-targeting therapies. Approaches to expand IL-35^+^ Bregs or IL-35^+^ Tregs *in vivo* has already been tested, such as through the provision of IL-12p35 (358, 370), or the heterodimeric cytokine (89), as well as MSCs overexpressing IL-35 (371). A novel strategy using IL-35^+^ Breg-derived exosomes that contain bioactive IL-35 has shown promising results as therapy in EAU; given the potential of exosomes to cross the blood–brain barrier, they could be considered for the treatment of autoimmune diseases affecting the central nervous system (372). Furthermore, studies of IL-35^+^ Tregs have demonstrated that this cytokine is displayed on the plasma membrane and associated with the tetraspanin molecule CD81 and that its release in exosomes enables transfer of surface IL-35 to bystander B and T cells (373). These results, in addition to the evidence of IL-35-dependent conversion of IL-35^+^ Bregs and IL-35^+^ Tregs, have prompted the proposal of an “infectious tolerance” mechanism that could amplify the therapeutic effect of IL-35-containing exosomes (374).

### Granzyme B

Bregs suppress inflammatory responses not only through the secretion of immunomodulatory cytokines but also through the release of cytotoxic GrB. Granzymes are a group of cytotoxic serine proteases that mediate target cell apoptosis upon entering the cytoplasm after perforin-mediated membrane disruption. There are 5 different types of granzymes in humans and there are 10 different types in mice. Among these, GrB has been described as the most powerful pro-apoptotic granzyme (375–377). Furthermore, GrB can also play a role in tissue remodeling by cleaving a number of components of the extracellular matrix and, in inflammation, through the processing of IL-1α, IL-18, and TGF-β (378–381).

Although GrB has been broadly described as part of the cytotoxicity machinery of activated CD8^+^ T cells and NK cells, Jahrsdörfer and colleagues described that BCR stimulation in the presence of IL-21, among other stimuli, can induce human B cells to secrete GrB and acquire cytotoxic potential. Several studies have shown GrB expression by other regulatory cell populations, such as Tregs and pDCs, which exert immunosuppressive functions over effector T cells, possibly mediated by perforin-independent degradation of the TCR ζ-chain, together with other mechanisms (382–384). Following these studies, an immunosuppressive role for GrB-secreting B cells was evaluated, observing that these cells are able to inhibit CD4^+^ T cell proliferation, as well as Th1 and Th17 responses, by a mechanism involving a rapid GrB-mediated degradation of the ζ-chain, but not T cell apoptosis (385, 386).

Moreover, GrB^+^ B cells are expanded in subjects vaccinated against viral diseases, implying a regulatory function in anti-viral immune responses (190, 385, 387). Indeed, CD4^+^ T cells from HIV patients that secrete IL-21 but express low levels of CD40L are able to expand a population of suppressive GrB^+^ Bregs (388). Although initially GrB-producing B cells were identified among stimulated naïve CD27^−^IgD^−^IgM^+^CD5^+^ cells, subsequent studies have also described other human B cell subsets with this capability, such as IgD^−^CD27^−^ double negative and memory B cells, as well as plasma cells (190, 389–391). It is important to consider that GrB expression has not been detected in murine B cells (190, 392).

Numerous studies have proposed a role for GrB^+^ Bregs in controlling inflammatory processes, since alterations in this population have been described in immune-related conditions. For instance, functional impairments in peripheral blood GrB^+^ Bregs were found in RA patients, which were reversed after achieving clinical remission (386). Likewise, patients receiving a kidney graft showed a decreased frequency of GrB^+^ Bregs, while an expansion of GrB^+^ Bregs was found in patients developing kidney transplant tolerance (390, 393). Contrastingly, it has been shown that unstimulated B cells from SLE, RA, and pSS patients exhibit a high expression of GrB (394–396), and GrB^+^ plasma cells were increased in the lamina propria of patients with inflammatory bowel diseases (397). This implies that, depending on the context, GrB^+^ Bregs can instead contribute to tissue damage secondary to autoimmune inflammation. Importantly, tumor- and lymph node-infiltrating GrB^+^ Bregs have been described in several carcinomas; however, whether GrB^+^ Bregs are important for anti-tumoral immunity or tumor evasion remains to be further elucidated (385, 398).

Although the abovementioned studies have confirmed the ability of B cells to secrete GrB to the extracellular milieu, a direct interaction with target cells has been proposed to be required for the inhibitory functions of GrB^+^ Bregs (385, 390). This contact dependency is not exclusive for GrB^+^ Bregs, as similar results have been described for IL-10^+^ Bregs (26, 265) and TGF-β^+^ Bregs (399), suggesting that cell-to-cell contact interactions are important either to promote Bregs to secrete modulatory molecules or as an independent mechanism of suppression.

## Suppressive Mechanisms of Bregs by Cell Surface-Expressed Molecules

**Figure 2:**
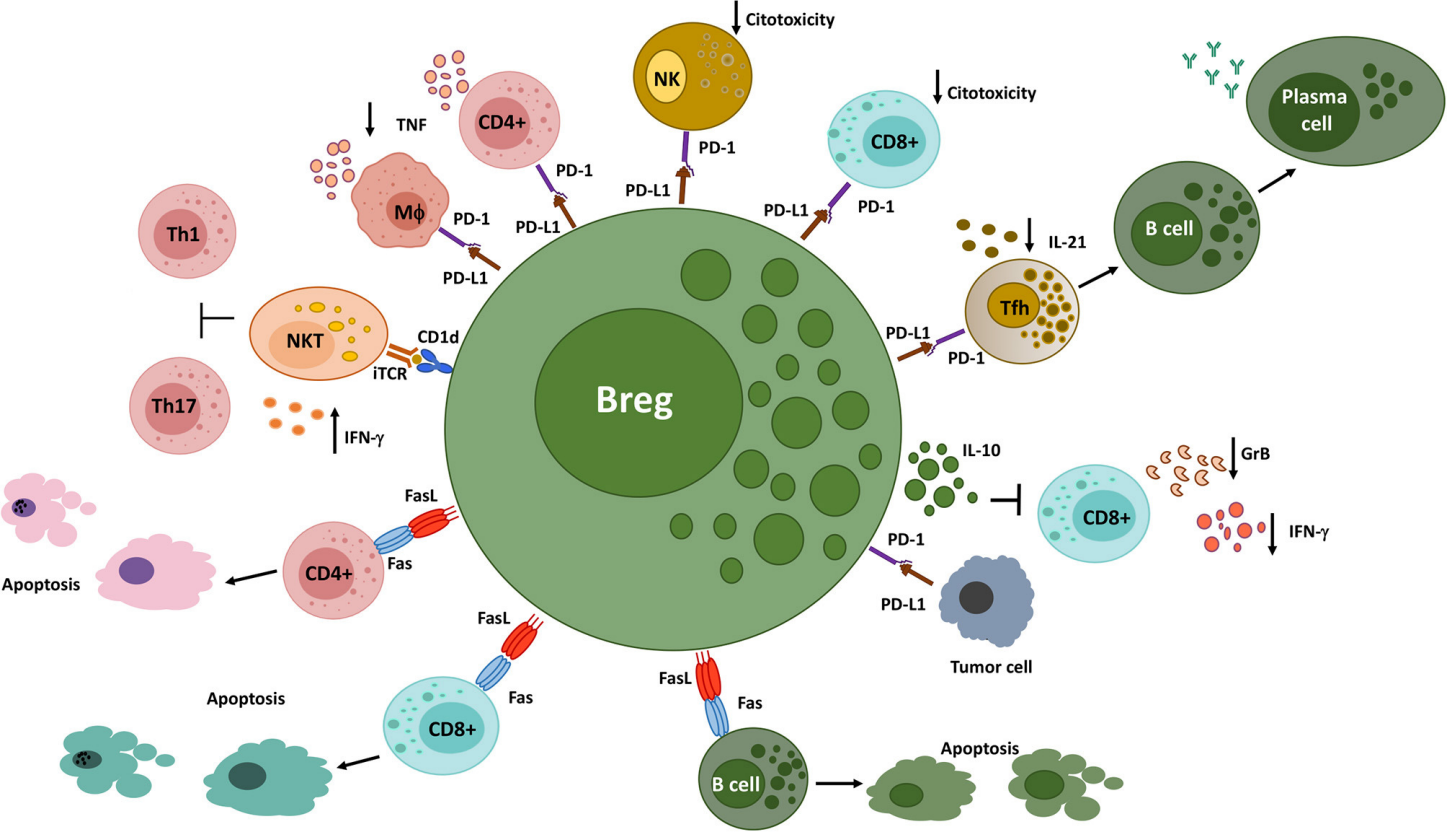
Different Breg populations utilize cell surface-expressed molecules to suppress immune responses. Bregs interact with NKT cells *via* CD1d, inducing the production of IFN-γ, which inhibits Th1 and Th17 responses in a murine arthritis model. PD-L1^+^ Bregs decrease the production of pro-inflammatory cytokines by PD-1-expressing CD4^+^ T cells and macrophages and the cytotoxic functions of PD-1-expressing CD8^+^ T cells and NK cells. In addition, they interact with PD-1-expressing follicular helper T cells, leading to an inhibition of humoral immune responses. On the other hand, PD-L1 expressed on tumor cells engage PD-1^+^ Bregs, triggering the production of IL-10 and the suppression of anti-tumor responses. Finally, FasL^+^ B cells induce apoptosis on Fas-expressing CD4^+^ and CD8^+^ T cells, as well as on B cells. GrB, Granzyme B; iTCR, invariant T cell receptor; MΦ, Macrophage; NK, Natural killer cell; NKT, Natural killer T cell; Tfh, Follicular helper T cell.

In the following section, we will discuss the suppressive mechanisms employed by Bregs through cell surface-expressed molecules (Figure 2), which can coexist or be autonomous from the soluble molecules examined above.

### CD1d

CD1d is a non-polymorphic MHC class I-like molecule that presents glycolipid antigens to a subset of T cells called natural killer T (NKT) cells. NKT cells are innate-like T cells that rapidly respond to glycolipid antigen recognition through their TCR, by secreting abundant amounts of cytokines that lead to the activation of CD1d-expressing cells, such as monocytes, macrophages, DCs, and B cells (400). NKT cells are divided into two populations based on their TCR diversity: Type I or invariant (i)NKT cells, which exhibit a semi-invariant TCR that recognizes the exogenous lipid antigen α-galactosylceramide (α-GalCer), and type II NKT cells that have a more diverse TCR repertoire (401). Interestingly, the administration of α-GalCer has shown to confer protection for a number of autoimmunity models, suggesting an immunoregulatory role for iNKT cells in certain conditions (402–404).

Among B cells, CD1d is most highly expressed on murine splenic CD24^hi^CD21^hi^CD23^lo^ MZ B cells and human MZ-like B cells and, to a lesser degree, by naïve and memory B cells. Once B cells present glycolipids to NKT cells *via* CD1d, activated NKT cells provide in return differentiating factors such IFN-γ and IL-4, allowing B cells to proliferate, mature, and secrete antibodies (405–408). *In vitro*, CD1d has been found to be upregulated shortly after CD40 activation (409). Early studies on different subsets of murine IL-10^+^ Bregs already described an upregulation of CD1d within these cells (4, 25, 29, 410). Moreover, CD1d expression on B cells was found to be essential for IL-10-dependent suppression of colitis (4). Similarly, human IL-10^+^ Bregs also express high levels of CD1d, as described for T2 transitional B cells, mature naïve B cells, and CD5^+^ B cells (249, 411, 412). CD1d^hi^ Bregs have also been shown to concomitantly secrete TGF-β in both mice and humans (87, 413). Despite these findings, a role for CD1d in mediating immune suppression by Bregs was not explored until recently.

Oleinika et al. demonstrated that α-GalCer fail to protect mice from arthritis in the absence of CD1d-expressing B cells. This protection was attributed to T2-MZP and was independent of IL-10 secretion by B cells. The authors of this study proposed a model where T2-MZP B cells would present α-GalCer *via* CD1d, inducing the secretion of IFN-γ by iNKT cells, which in turn suppress Th1 and Th17 responses in arthritis (414). Although this study ruled out a requirement of iNKT cells for IL-10^+^ Breg differentiation, previous findings have suggested that cognate interactions with iNKT cells can drive an expansion of Bregs (415, 416). Likewise, abnormalities in iNKT cell homeostasis have been attributed to dysfunctional CD1d-mediated presentation of self-lipids by B cells in autoimmunity-prone mice, thus confirming the importance of CD1d^hi^ B cells in maintaining self-tolerance (417).

In line with these results, several studies have observed alterations in the NKT compartment in patients with autoimmune diseases such as RA, SLE, and MS (418–421). Although the causes and consequences of these alterations are not fully understood, it has been shown in healthy individuals that B cells are essential for *in vitro* iNKT proliferation, activation, and cytokine production in a CD1d-dependent fashion, a pathway that is defective in SLE patients. This defect could be explained by a decrease in CD1d surface expression on SLE transitional B cells due to a higher internalization rate (412).

### PD-1/PD-L1

Immune checkpoints are inhibitory receptors that modulate the activation of immune cells in order to limit immune responses and preserve self-tolerance. The importance of immune checkpoints is highlighted not only by the success of therapeutic approaches blocking CTLA-4 and PD1 that boost anti-tumor responses in cancer patients but also by the autoimmune side effects unleashed by these drugs (422, 423). Experience from checkpoint blockade has inspired the design of therapies to activate immune checkpoints for the treatment of autoimmune diseases (424). Among these, there are increasing studies demonstrating the efficacy of PD-1 activating therapies in mouse models of EAE, colitis and lupus, as well as in transplantation (425–429).

PD-1 is a type I transmembrane receptor expressed in activated monocytes, DCs, NKT cells, B cells, and T cells. The engagement of PD-1 by its ligands, PD-L1 and PD-L2, delivers inhibitory signals that downmodulate receptor-triggered cell survival, differentiation, and secretion of pro-inflammatory cytokines. PD-L1 is constitutively expressed on mouse T cells and B cells, DCs, and macrophages, among other cell types, while PD-L2 expression is restricted to mature DCs, macrophages, mast cells, and a subset of B1 cells. PD-L1 and PD-L2 can also deliver reverse inhibitory signals upon PD-1 or CD80 engagement. Of note, both PD-1 and PD-L1 are highly expressed on Tregs and have been involved in Tregs induction and suppressive functions (430–433). These findings prompted investigations to discover similar functions of PD-1 and its ligands in Breg biology.

Studies have shown that some PD-1^+^ and PD-L1^+^ B cells co-express IL-10 and, that upon engagement of PD-1, suppress CD4^+^ and CD8^+^ T cell activity and induce Tr1 cells, suggesting a role of PD-1 in promoting IL-10 expression (292, 296, 434, 435). For instance, CD5^hi^PD-1^+^ B cells with a memory phenotype have been found to be enriched in hepatocellular carcinoma and to produce IL-10 upon PD-1 engagement by PD-L1 (436). Adoptive transfer of CD5^hi^PD-1^+^ B cells from hepatoma-bearing mice effectively suppressed CD8^+^ anti-tumor responses and promoted tumor growth (436). Another study reported that PD-L1 on human tumors can endow tumor-infiltrating B cells with Treg-inducing properties (437). Also, human PD-1^+^PD-L1^high^ Bregs infiltrating thyroid tumors were shown to decrease CD4^+^ and CD8^+^ T cell survival, an effect that was reversed by PD-L1 blockade (438). Nevertheless, other studies in cancer have shown that PD-L1 can mediate IL-10-independent suppression by B cells. In pancreatic cancer models, PD-L1^+^ B cells were reported to inhibit NK and CD8^+^ T cell cytotoxicity *via* PD-L1 (359, 439). PD-L1^+^ Bregs have also been described to be expanded in tumors and draining lymph nodes of mice bearing breast or cervical cancer (399, 440, 441). In addition, PD-L1^+^ B cells have been identified within IgA^+^ B cells in mice bearing liver tumors and have been proposed to mediate resistance to chemotherapy in prostate cancer, which can be overturned by PD-L1 blockade (50, 442). Altogether, these findings underscore a novel role of Bregs behind the mechanisms of action PD-1/PD-L1 targeting therapies in cancer.

IgA^+^ plasma cells from small intestine lamina propria require both PD-L1 and PD-L2 to induce Tregs, suggesting that PD-1/PD-L1^+^ Bregs could be important in keeping peripheral tolerance (54). Along these lines, IgA^+^PD-L1^+^ Bregs were shown to dampen TNF production by macrophages and T cells and to ameliorate EAE development (61). Other PD-L1^+^ B cell subsets have also been shown to be essential for EAE protection by reducing Th1/Th17 responses (443–445). It has also been described that PD-L1-expressing Bregs have high avidity for BAFF and that these cells are spared after B cell depletion in EAE mice, an intervention that raises BAFF serum levels, revealing a novel aspect of a therapy that is successfully used in patients with MS (446). Furthermore, alterations in PD-L1^+^, PD-L2^+^, and PD-1^+^ Bregs have been reported in patients with autoimmune conditions (237, 434, 447–450).

Inflammatory signals also trigger the upregulation of PD-L1 in B cells (434, 451). It has been described that PD-L1 expression on B cells is crucial in supporting the generation of long-lived plasma cells, limiting the expansion of PD-1-expressing Tfh cells but increasing the availability of IL-21 (452–455). However, a recent report showed that the adoptive transfer of PD-L1^hi^ B cells to EAE mice results in milder disease associated with a reduced Tfh-cell expansion, as well as decreased antigen-specific IgG and Th1/Th17 cells (446). These findings suggest that, during inflammation, expression of high levels of PD-L1 can endow B cells with regulatory properties. Finally, PD-1 and PD-L1 expression on B cells was described to be upregulated by *in vitro* stimulation with *Helicobacter* and to mediate Tr1 differentiation (456). Moreover, PD-L1 upregulation on B cells, associated with T cell exhaustion, was observed in RSV-infected mice (457) and HIV-infected patients (455), suggesting a role for PD-L1^+^ Bregs as a mechanism to limit tissue damage that can be subverted by pathogens in their benefit.

### FasL (CD95L)

Above, we have discussed the inactivation of immune cells through the secretion of GrB as one of the suppressive mechanisms displayed by Bregs. However, induction of anergy or apoptosis of activated T cells by Bregs can also rely on cell surface-expressed molecules (458). Ligation of the Fas death receptor (CD95) on activated T cells by FasL, a process known as activation-induced cell death, is a common apoptotic pathway. The Fas:FasL interaction is essential for the maintenance of self-tolerance, as alterations in the expression of these molecules lead to spontaneous systemic autoimmunity in mice (459). Remarkably, selective deletion of FasL on B cells is able to break tolerance and drive T cell expansion and the production of autoantibodies (460). Expression of functional FasL in murine B cells has been described upon activation by LPS plus P+I (461). In addition, FasL-expressing human B cells have been identified in tonsil GCs, in the bone marrow, and among plasma cells populating different tissues (462–464). Killer B cells constitutively expressing FasL were found to be expanded in autoimmune-prone mice, a finding that was also reported in peripheral blood of SLE and type 1 diabetes patients (465–467). Whether this expansion is due to compensatory mechanisms and/or contributed to the pathogenesis of autoimmune diseases is yet to be resolved; however, progress has been made in recent years.

The physiological relevance of FasL^+^ B cells was assessed in the NOD mouse model of diabetes, where these cells promote apoptosis of diabetogenic T cells *in vitro* and a reduction of antigen-specific Th1 responses *in vivo* (468). FasL^+^ B cells were also shown to directly mediate apoptosis of CD4^+^ T cells in a murine model of *Schistosoma* infection (469). During *Schistosoma* infection, constitutively high expression of FasL was found within splenic CD5^+^ B cells (470). Moreover, in arthritic mice, splenic CD5^+^FasL^+^ B cells reduced the frequency of pathogenic Th17 cells in a FasL-dependent manner (471). It was also shown that FasL^+^ B cells fall into the CD5^+^CD1d^hi^ population, and there was only partial overlap with IL-10^+^ Bregs (472, 473). Another population of FasL^+^ Bregs have been described within immature IgM^−^CD1d^+^ pro-B cells, which are able to kill effector T cells while protecting NOD mice from diabetes (474).

FasL^+^ Bregs can also be subverted by infectious agents to avoid inflammatory responses. FasL^+^ Bregs have been detected upon *in vitro* infection with Epstein–Barr virus and in patients with filarial parasites or HIV infections (475–477). In these circumstances, FasL^+^ Bregs can also induce the apoptosis of cytotoxic CD8^+^ T cells, which can correlate with a reduced control of the infection (478, 479). The targets of FasL^+^ B cells are not restricted to T cells, as shown in a murine model of *Trypanosoma* infection, where a “fratricide” killing of parasite-specific B cells has been described (480, 481). Furthermore, in an inflammatory context, FasL^+^ B cells can also induce apoptosis of non-immune cells, such as pulmonary epithelial cells in an acute lung injury model (482). Besides, FasL^+^ B cells recovered from tumor-draining lymph nodes were capable of killing tumor cells *in vitro* (483, 484). Taken together, these results show that FasL^+^ Bregs are generated under inflammatory conditions and that they may play a role in the maintenance of peripheral tolerance and control of exacerbated responses but can also be responsible for inflammation-induced damage and anti-tumoral immunity.

It is worthwhile noting that B cells have been reported to induce contact-dependent apoptosis or immune modulation through other members of the TNF family, such as membrane-bound TNF (485), GITR (486), and TRAIL (487, 488). In addition, recent studies have shown that IL-10^+^ Bregs from RA patients can acquire ectopic expression of RANKL, an important molecule involved in osteoclast activation and bone destruction (489).

## TIM-1-Expressing Bregs (TIM-1^+^ Bregs)

TIM-1^+^ Bregs are a newly discovered population of human and murine Bregs that has gained increasing attention, since TIM-1^+^ Bregs phenotypically and functionally overlap with many of the abovementioned subsets, including IL-10^+^ Bregs, TGF-β^+^ Bregs, and PD-L1^+^ Bregs. TIM-1 can function as a receptor that licenses B cells to express suppressive cytokines or ligands, although, a contact-dependent effect on immune cells expressing TIM-1-binding partners has not been excluded so far.

TIM receptors represent a family of molecules that play critical roles in the regulation of immune responses. To date, four members of this family have been identified in mice (TIM-1,−2,−3,−4) and three in humans (TIM-1,−3, and−4) (490). TIM-1 is expressed in several immune cells, including activated T cells, Th2 cells, B cells, NKT cells, macrophages, and DCs, whereas TIM-4, one of the ligands for TIM-1, is expressed on monocytes, macrophages, and DCs (491). TIM-1 has been described as a phosphatidylserine (PS) receptor mediating phagocytosis of apoptotic bodies and in the regulation of immune responses (492). In T cells, TIM-1 functions as a co-stimulation signal, inducing T cell activation and IL-4 secretion (493, 494). In DCs, TIM-1-mediated signaling promotes their maturation, enhancing their ability to induce Th17 responses and inhibit the generation of Tregs, implying a cell-dependent fine-tuning of TIM-1 functions (495).

Early studies identified TIM-1 expression in murine GC B cells (496, 497). Later on, it was shown that mice treated with a low-affinity agonistic anti-TIM-1 antibody, either alone or in addition to anti-CD45RB, displayed long-term tolerance to allografts, an effect that was completely reversed in the absence of B cells. B cells were shown to be responsible for anti-TIM-1-mediated Th2 responses and IL-10 production by Tregs in these settings. TIM-1^+^ B cells were enriched in IL-4- and, importantly, TGF-β- and IL-10-producing cells, regardless of the developmental stage or anatomic localization of these populations and were induced and expanded after transplantation or anti-TIM-1 treatment in grafted mice. Both IL-10 and TGF-β have been found to be essential for antigen-specific tolerogenic properties of adoptively transferred TIM-1^+^ Bregs (52, 91, 498–500). The regulatory nature of TIM-1^+^ B cells was further validated in different models, as the transfer of these cells can reduce the severity of allergic airway inflammation (91) and colitis (501), while promoting tumor growth (183, 502). These results support a role of TIM-1 as an encompassing IL-10^+^ and TGF-β^+^ Breg marker with functional implications.

Subsequently, it was demonstrated that the mucin domain of TIM-1 was crucial for IL-10 induction by B cells following stimulation with PS-containing apoptotic cells, suggesting a physiologic pathway whereby dying cells promote tolerance to self-antigens and resolution of inflammation by activating IL-10^+^TIM-1^+^ Bregs (503–505). This proposition is further sustained by observations in mice with a B cell-specific deletion of TIM-1 or mice lacking the mucin domain of TIM-1, which present a profound defect in IL-10^+^ Bregs and multi-organ tissue inflammation. Furthermore, these mice exhibit a severe EAE course or accelerated lupus-like syndrome on a susceptible background (506, 507). Moreover, amelioration of EAE following inoculation with apoptotic cells was abrogated in recipient mice with TIM-1-deficient B cells, showing expanded Th1/Th17 responses and reduced Treg generation (508).

Accordingly, *in vitro* and *in vivo* studies have demonstrated that murine TIM-1^+^ Bregs are able to inhibit the differentiation of Th1 or Th17 cells and to promote Treg and Tr1 generation, which has been primarily attributed to the production of IL-10 or TGF-β, although cytokine-independent mechanisms have also been suggested (91, 183, 500, 507–510). In this regard, a recent study has revealed that the expression of the immune checkpoint TIGIT is enriched among TIM-1^+^ Bregs and depends on TIM-1 signaling and subsequent activation of AhR. TIGIT-deficient B cells showed an impaired IL-10 production, facilitating the development of spontaneous neuroinflammation with infiltration of Th1 and Th17 cells. In addition, these mice presented a more severe EAE, while transfer of TIM-1^+^ B cells from TIGIT-deficient mice showed reduced ability to ameliorate EAE. Since not all TIGIT^+^ B cells express IL-10, and vice versa, these results suggest that TIGIT expression can be an independent mechanism of suppression by TIM-1^+^ Bregs (507).

A number of reports showing the expression of TIM-1 in human B cells have been published in recent years. In accordance with mouse data, studies in healthy donors and HIV patients found an enrichment of IL-10-producing cells within TIM-1^+^ B cells. In patients with HIV infection, TIM-1^+^ B cells specific for HIV antigens suppressed IL-17 and IFN-γ production by HIV-specific CD8^+^ and CD4^+^ T cells, which was partially reversed by IL-10 blockade (293, 511). Human IL-10^+^ TIM-1^+^ Bregs can also be found infiltrating tumors and have been described to suppress CD8^+^ and CD4^+^ T cell anti-tumor responses (323, 512, 513). The utility of TIM-1 as a marker for a proportion of human IL-10^+^ Bregs has been further confirmed in inflammatory and autoimmune diseases, such as in RA patients, who exhibit reduced frequencies of TIM-1^+^ Bregs (514). In agreement with these studies, we reported that TIM-1 and IL-10 were preferentially co-expressed in transitional B cells from healthy donors upon BCR and TLR9 activation; however, this population is significantly decreased in patients with systemic sclerosis (SSc). In addition, TIM-1^+^ Bregs from SSc patients exhibited an impaired suppression of pro-inflammatory cytokines by CD4^+^ T cells (515). As for mice, human TIM-1^+^ Bregs have also been proven to secrete TGF-β, together with IL-10, and to promote Tregs differentiation, which seems to be mediated by TGF-β (510, 516).

**Figure 3:**
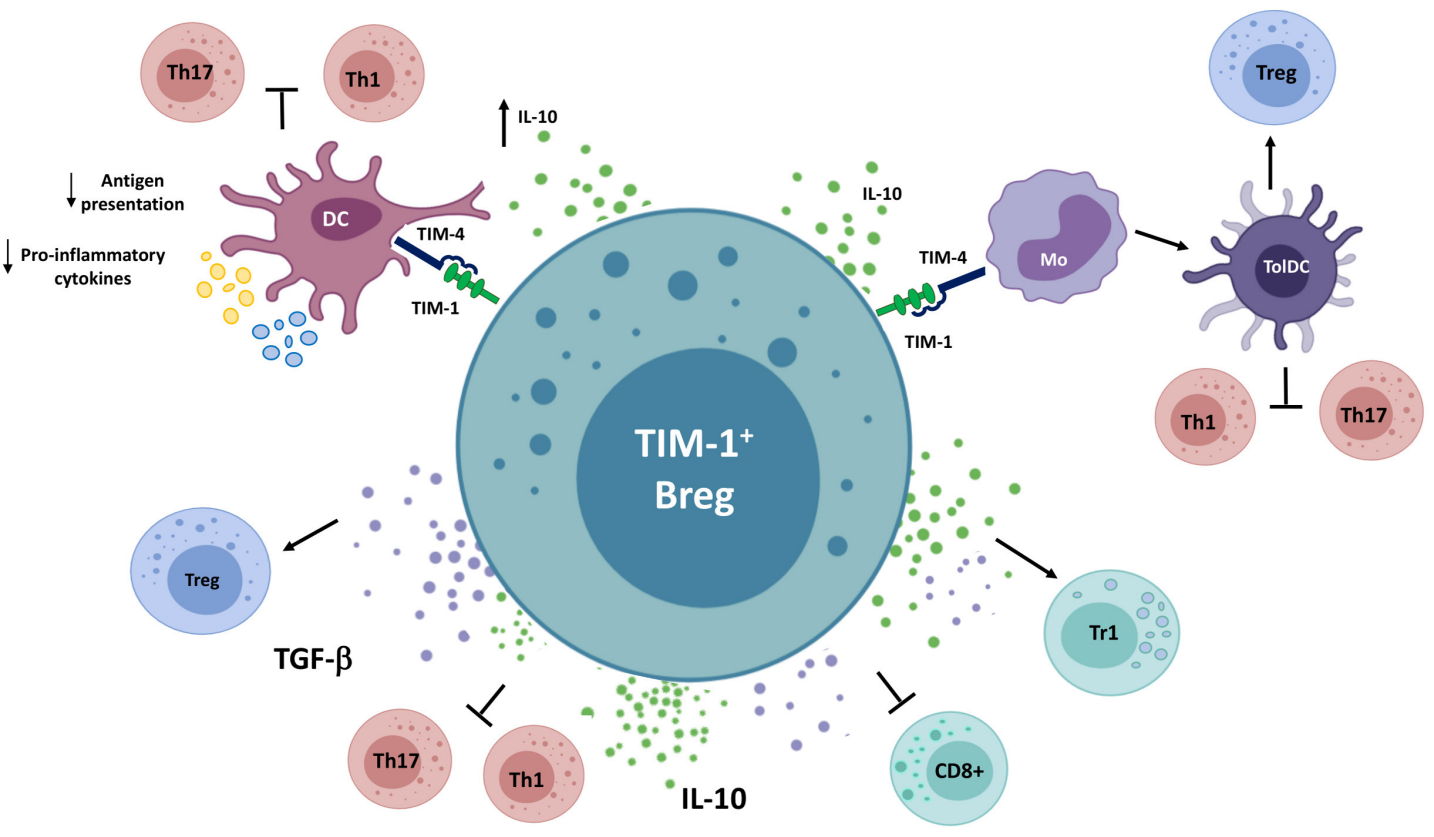
Model of suppression of immune responses by TIM-1^+^ Bregs. TIM-1^+^ Bregs secrete IL-10 and TGF-β upon engagement of TIM-1 by phosphatidylserine and possibly by interacting with TIM-4-expressing antigen-presenting cells, inhibiting Th1, Th17, and CD8^+^ T cells, as well as inducing regulatory T cell populations. These changes could also be mediated indirectly, by decreasing antigen presentation and the production of pro-inflammatory cytokines by dendritic cells (DCs) or modulating monocytes to differentiate into tolerogenic DCs. DC, Conventional dendritic cell; Mo, Monocyte; TolDC, Tolerogenic dendritic cell; Tr1, Type-1 regulatory T cell.

Therefore, it is becoming apparent that TIM-1^+^ Bregs have a regulatory role in the homeostasis of the immune system, but more studies are necessary to unveil the mechanisms that this population employs for such effects. Currently, our group is characterizing the influence of TIM-4 and TIM-4-expressing cells in the activation of TIM-1^+^ Bregs. Previous studies using a fusion Fc-TIM-4 murine protein have shown that TIM-4 binds to and activates TIM-1 expressed on T cells (490, 517, 518). Human and murine TIM-4 are expressed in APCs, recognize PS, and are important for the clearance of apoptotic cells (490, 519, 520). This has led to the interpretation that TIM-1:TIM-4 interactions could be bridged by PS-containing exosomes (519, 521). Additionally, TIM-4^+^ myeloid cells have been encountered infiltrating human tumors in close vicinity to TIM-1^+^ B cells, which suggests that myeloid cells could induce TIM-1^+^ Bregs to produce IL-10 through TIM-1:TIM-4 interactions (513). We have preliminary data indicating that TIM-1^+^ Bregs directly interact with TIM-4^+^ APCs and decrease their antigen presentation capacity, as well as the production of pro-inflammatory cytokines. From these data, and previous evidence, we postulate that TIM-1^+^ Bregs could be involved in tolerance induction both directly acting on T cells and indirectly by modulating TIM-4-expressing APCs or generating tolerogenic DCs, which result in the suppression of pro-inflammatory T cells and the induction of Tregs (Figure 3).

## Concluding Remarks

The crucial role of Bregs in homeostasis and different immune conditions is now emerging in the literature, as new evidence confirms them as an important member of the immunosuppressive cell family. However, much needs to be elucidated regarding their origin, phenotype, function, and suppressive mechanisms. Experimental studies have focused on soluble mediators as the main regulatory molecules of Bregs. On the other hand, research on surface molecules that allow Bregs to establish cellular interactions with other cell populations and convey inhibitory signals is lagging behind. Herein, we have aimed to consolidate and update the current knowledge about the cellular and molecular regulatory mechanisms that are exhibited by Bregs and identify the main cell populations targeted by them. It is conceivable that the considerable phenotypical and functional Breg heterogeneity is required to effectively limit inflammation and reestablish immune homeostasis in diverse anatomical sites, stages of the inflammatory process, and pathological conditions. Understanding how Bregs are able to exercise their suppressive action will be of great importance when applying the biological function of Bregs in clinical settings.

## Author Contributions

DC, OA, MAM, AF, KO, JCA, and LS discussed the contents, wrote, reviewed, and edited the manuscript. All authors listed approved the version to be published and have made a substantial and intellectual contribution to the work.

## Conflict of Interest

The authors declare that the research was conducted in the absence of any commercial or financial relationships that could be construed as a potential conflict of interest.
